# Mindfulness-based stress reduction intervention for elementary school teachers: a mixed method study

**DOI:** 10.1186/s13063-021-05804-6

**Published:** 2021-11-21

**Authors:** J. H. Lensen, S. E. M. J. Stoltz, M. Kleinjan, A. E. M. Speckens, J. T. Kraiss, R. H. J. Scholte

**Affiliations:** 1 Rotterdamse Vereniging Katholiek Onderwijs, Rotterdam, The Netherlands; 2 Behavioural Science Institute, Radboud University, Nijmegen, The Netherlands; 3 Trimbos Institute (Netherlands Institute of Mental Health and Addiction), Utrecht, The Netherlands; 4 Interdisciplinary Social Science, Utrecht University, Utrecht, The Netherlands; 5 Faculty of Medical Sciences, Radboud University Medical Centre, Nijmegen, The Netherlands; 6 Department of Psychology, Health, and Technology, University of Twente, Enschede, The Netherlands

**Keywords:** Mindfulness, Elementary school teachers, Stress, Teacher skills, Mental health

## Abstract

**Background:**

In the Netherlands, more than half of the teachers working in primary education experience high levels of work stress. Compared to other professions, teachers are more likely to drop out from work and develop mental illnesses. Almost one in five even choose a new profession within 5 years after starting as a teacher. This indicates an urgent need for interventions to reduce stress levels in teachers. However, few evidence-based effective interventions targeting stress and work-related problems in the primary educational system are available.

**Aim:**

In the current paper, we describe the protocol for a randomized controlled study (RCT) comparing an 8-week mindfulness-based stress reduction (MBSR) intervention with a wait list control condition in primary school teachers. We hypothesize that teachers who participate in the MBSR programme will report less stress (primary outcome) than those in the control group at post-test and at 3-month follow-up. We also expect a decrease in teachers’ absenteeism and improvements of mental health, teacher skills, classroom climate quality and the pupil-teacher relationship (secondary outcomes). Finally, we hypothesize that self-compassion, mindfulness skills and emotion regulation skills could mediate effects.

**Methods/design:**

A mixed-method study will be conducted among *N*=155 Dutch primary school teachers (grade 1 to 6). The quantitative study will be an RCT, in which teachers will be randomly allocated to the MBSR or waiting list control condition. Trial participants will not be made actively aware of their condition. The data analysts will be blinded. Online questionnaires will be sent to teachers before and after the MBSR programme, and at 3-month follow-up. Information about absenteeism will be collected. In the qualitative part of the study, we will interview teachers to examine their perceived effects of MBSR on their teaching skills, the classroom climate quality and the pupil-teacher relationship.

**Discussion:**

This protocol paper describes a mixed-method study design with an RCT and a qualitative evaluation to evaluate an MBSR programme on perceived stress among primary school teachers. If the MBSR programme proves to be effective, it could be implemented as a programme to reduce stress and improve mental health and teaching outcomes in primary school teachers.

**Trial registration:**

Nederland Trial Register NL. Registered on 19 November 2019—retrospectively registered, https://www.trialregister.nl/trial/8171

## Background

In recent years, teachers in primary education report increasing levels of work pressure. For more than half of the teachers (i.e. 56%) in the Netherlands, this work pressure is unacceptably high [1]. This pressure is not only related to the quantity of work, but also to the emotional demands of the work [2]. According to the Transactional Model of Stress and Coping (Lazarus, 1991 [3];), experienced stress is the result of the interpretation of a stressor and the capacity to regulate emotions. The continued experience of negative emotions in reaction to external stressors can cause feelings of stress [4]. Continuing high levels of stress can, in turn, lead to absenteeism or premature outflow from the profession in which the stress is experienced. In the Netherlands this is reflected in a high percentage of absenteeism and burnout symptoms in primary school teachers [5]. Furthermore, figures show that of all starting teachers, one in five leave the Dutch educational system within 5 years. One in eight teachers is actively searching for employment outside the educational field. Work pressure and related stress are specified as the most important reason [6].

Continued stress in teachers leads to adverse effects on their mental health and well-being. Roffey [4] conducted research in six Australian schools and showed that the extent to which teachers can cope with stress and regulate their emotions influences both cognitive and social performances. Stress and coping with stress also affect so-called teacher skills: skills needed to be capable of delivering high-quality education and establishing effective class management [7]. A review by Spilt et al. [8], in which results of 99 studies have been analysed, showed that stress in teachers had a negative impact on the pupil-teacher relationship. In turn, a poor pupil-teacher relationship had both direct and indirect adverse effects on the teacher’s mental health and wellbeing [3, 9]. It also led to misbehaviour, lack of motivation and diminishing achievements in pupils [3, 4, 8], which may also contribute to perceived teachers’ stress [2].

Even though there seems a great need for alleviating stress in teachers, studies exploring ways to do so in primary schools are limited [2]. A meta-analysis of 65 independent studies on teachers’ stress showed that improved emotion regulation could be the key to stress reduction [2]. Only 17 of these studies concerned primary school teachers. However, research shows that primary school teachers are emotionally more involved in their teaching than teachers in higher education [10]. Unlike teachers in higher education, primary school teachers teach their pupils for 5 days a week. Skills such as insight into their own emotions and thoughts and the capacity to regulate these effectively while teaching, might contribute to reduction of perceived stress. Teachers who possess these skills are able to build supporting relationships with their pupils more effectively, experience more positive emotions, possess more mental resilience and experience more job satisfaction [2].

Besides the influence of teachers’ experienced stress on their own well-being and functioning as a teacher, this stress may ultimately affect their pupils as well [11]. Briner and Dewberry (2007) have found indications that the level of well-being of the teacher affected the classroom climate. A physically and socially safe classroom climate is a necessary condition for pupils to be able to learn, and classroom climate directly affects the well-being and performance of pupils [3, 12–14]. In addition to the development of didactic and organizational qualities, the development of a good pedagogical relationship with pupils is therefore of great importance [4]. Providing teachers with means to alleviate stress and improve their mental health and well-being is thus not only in the interest of teachers themselves, but also in the interest of their pupils.

### Mindfulness-based stress reduction (MBSR)

A promising method to improve emotion regulation and reduce stress symptoms is mindfulness-based stress reduction (MBSR) as developed by Jon Kabat-Zinn (2004). Kabat-Zinn defines mindfulness as ‘Being aware in a special way: be consciously present in the here and now, without judgment’. Mindfulness can contribute to reducing stress by gaining insight in behavioural patterns, creating emotional awareness, and develop regulation of emotions [15].

A meta-analysis of 29 studies on effectiveness of mindfulness interventions in healthy adults showed a reduction of experienced stress, depressive and anxiety symptoms and improvements of quality of life [16]. The results of a review and meta-analysis including over 12,000 people with psychiatric disorders such as depression demonstrated that mindfulness-based interventions were as effective as other evidence-based treatments including cognitive behavioural therapy and anti-depressant medication [17]. An article by Tang et al. [15] specifically confirmed the positive effect on the regulation of emotions. Other studies showed promising effects of MBSR on worrying, regulation of attention and emotions, personal achievements and empathy [7].

Although there are promising effects of mindfulness on stress and mental health in diverse populations, only few studies have been conducted to date on the effectiveness of mindfulness in primary school teachers. Most of these studies have been conducted in the USA. European studies are generally lacking, and no studies on this subject have been conducted in the Netherlands.

Jennings and colleagues’ research (2017) in 224 American K-5 grade teachers is the largest RCT to date that examines the effectiveness of a mindfulness-based intervention (MBI) for teachers in primary education. In contrast to the control group, the intervention group displayed significant reduction of stress and perceived time pressure. In addition, teachers in the intervention group reported an improvement of mental health and well-being. They experienced more self-awareness, mindfulness (i.e. observing without judgement) and better regulation of emotions. They reported to be able to deal with their pupils’ behaviour more effectively and more compassionately. This is reflected in the fact that 91% of the teachers in Jennings’ study reported to be more capable of starting and maintaining a supporting relationship with their pupils. The study by Jennings and colleagues delivers the most compelling evidence so far that MBIs can contribute positively to stress experience, mental health, executive functions, teacher skills, the classroom climate and the pupil-teacher relationship in primary school teachers.

Earlier studies in teachers from various forms of education [18–20] showed that when teachers can deploy mindfulness skills, they were more capable of assessing emotionally provoking situations in the classroom and reacting adequately. This resulted in less stress and more mental well-being. This way, mindfulness skills positively affected the interaction with pupils and the pupil-teacher relationship.

Because of the lack of studies outside the USA, we conducted an uncontrolled pilot study to examine the potential of an MBSR programme within 71 primary school teachers. After the training, participants showed a significant reduction of perceived stress (PSS, Perceived Stress Scale) and improvements of psychological, emotional and social well-being (MHC-SF, Mental Health Continuum-Short Form). In addition, the pilot study showed a significant increase in both mindfulness skills (FFMQ-SF, The Five Facet Mindfulness Questionnaire Short Form) as well as self-compassion (SCS-SF, Self-Compassion Scale-short Form), both of which are related to mental health. In addition, we tested several potential moderators and found indications for possible moderating effects of school weight and current or past psychological problems on the impact of the MBSR programme. Because of these promising results we will test the effectiveness of MBSR in Dutch primary care teachers on larger scale including a control group by conducting an RCT.

### Aims of the trial

The primary aim of this study is to investigate the effectiveness of MBSR in contrast to a wait list control condition in reducing perceived stress in teachers in primary education. The secondary aim is to examine the effects of the MBSR on (a) absenteeism of teachers, (b) teachers’ mental health, (c) teacher skills and (d) teachers’ experienced classroom climate and pupil-teacher relationship.

The third aim is to examine possible mediating factors of treatment outcome, such as mindfulness skills (i.e. observing, describing, acting with awareness, non-judgement and non-reactivity), self-compassion and emotion regulation skills. Based on research by Jennings [2], Roeser [18], Roeser et al. [19] and Skinner and Beers [20], we hypothesize that an improvement of mindfulness skills, self-compassion and emotion regulation skills will lead to a reduction of perceived stress. In addition, we explore whether school weight, age, past and present psychological problems and teachers’ years of experience act as potential moderators of programme effects. The final aim is to conduct qualitative interviews after the MBSR programme to get a more in-depth view of teachers’ perception of the impact of MBSR on their teacher skills, classroom climate and pupil-teacher relationship.

## Methods/design

### Study design

**Fig. 1:**
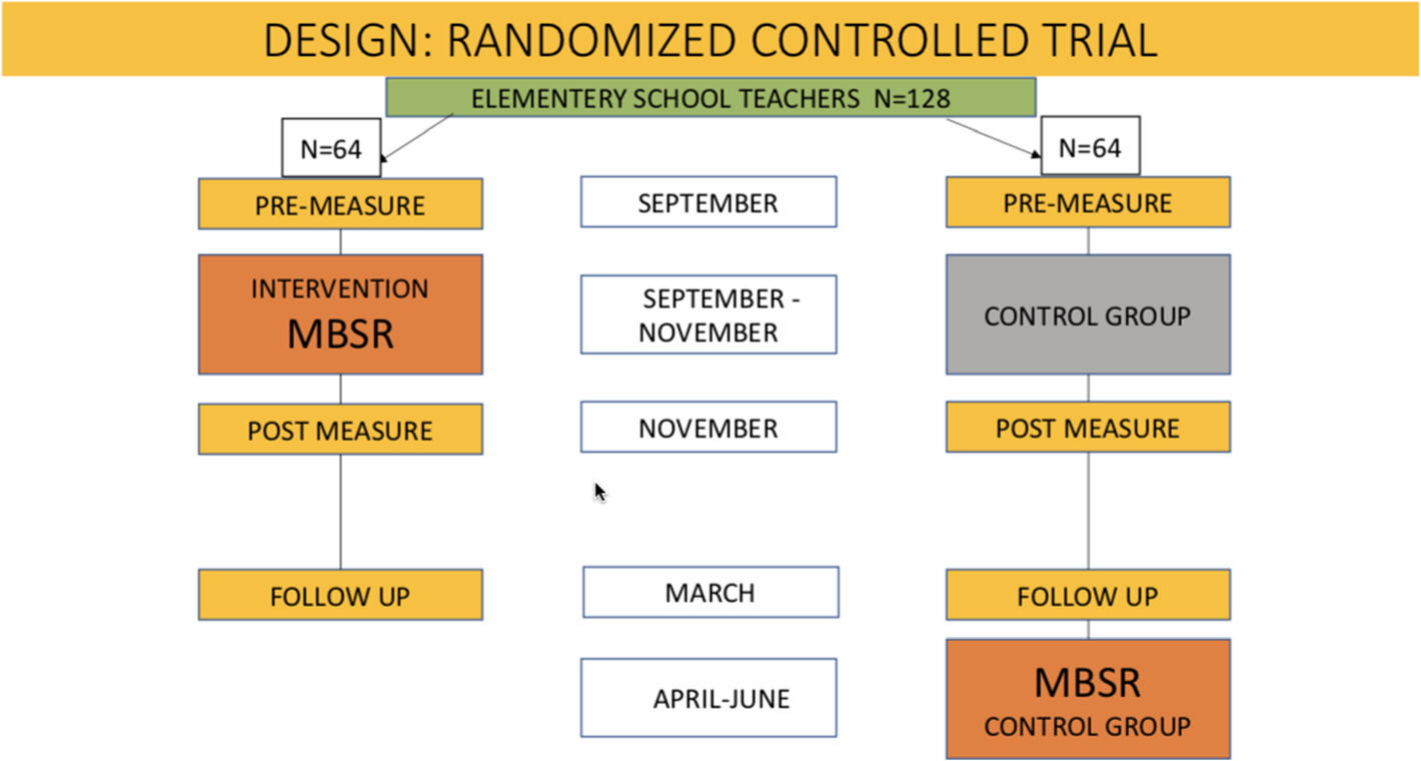
Flow diagram of the study design

The design of the study is a superiority RCT in which primary education teachers are randomly allocated with a ratio of 1:1to either the MBSR programme or the waiting list control condition (see Fig. 1). Because of practical and financial constraints, the control condition consists of no intervention. As we would like to offer MBSR to all the teachers who express interest, we also wanted to be able to offer it to those allocated to the control condition. The study protocol has been ethically approved by the Internal Review Board (IRB) of the [masked for blind review] and is registered under number ECSW-2019-029.

### Participant characteristics

The study population will consist of Dutch teachers in primary education teaching groups 1–8, comparable to grades 1 to 6 in the US school system.

#### Eligibility criteria

We will include teachers who meet the following criteria: (a) teaching at least two days in the same group during a school year between the start of the school year and March, (b) willing to fill out questionnaires in Dutch three times in a school year and (c) able to start the programme in either September or March.

Exclusion criteria will be previous participation in a Mindfulness-Based Cognitive Therapy, MBSR programme or a mindfulness-based workshop of more than 3-h duration. During the trial, there will be no restrictions with regard to concomitant interventions. However, participants will be requested to not participate in an MBSR course offered elsewhere if they are allocated to the wait list control condition.

### Procedure and allocation

Recruitment will take place in a large urban area in the Netherlands. School boards in the study area will be informed about the study and asked to make their teachers aware of it. Teachers will also be recruited by social media and by presentations at schools. Interested teachers will receive an information leaflet and be invited for a screening interview by telephone. Teachers who are willing to participate will provide written informed consent which will be obtained by one of the researchers (JHL).

Participants will receive online questionnaires, which will be processed anonymously. Questionnaires will be administered before and after the MBSR or wait list period (September and November), and at 3 months’ follow-up (March). As participants will be aware whether they have or have not participated in the course by the time they complete the follow-up assessments, they cannot be kept completely blinded. We will compare data related to teacher absenteeism from the school year prior to that in which they participate in the research. After completion of the follow-up, teachers of the control group will be offered the MBSR programme as well. To include the required 155 participants, the intervention procedure between September and June will be repeated for three consecutive school years.

The Ethics Committee Social Science (Radboud University) agreed (ECSW-2019-029) that there was no risk of physical or mental harm for participants during this trial. The Faculty of Social Science takes the juridical responsibility for accidents and damage caused by participating in the research. If participants report any adverse events during the trial, either during the MBSR course or during the wait list control condition, this will be registered in the electronic database system Castor. In case of serious adverse events, these will be reported to the Ethics Committee. If participants require additional mental health support, they will be referred to the regular services, but it is also possible for participants to consult with an appointed psychologist if they so request, or if the trainer so advises. This psychologist collaborates with the overarching school organization/foundation to which most of the participating schools and therefore teachers in this study belong. If recruited participants do not attend the MBSR course, they will be contacted by the MBSR teacher. If they decide to discontinue, they will be given suggestions on how to obtain mental health support if needed. Lastly, if participants have questions about the study or the planning, they can always contact one of the researchers whose contact details are listed in the consent letter. These details also include the contact details of an independent supervisor at the Radboud University who can be contacted in case of complaints.

### Randomization

Randomization will be performed after completion of the informed consent forms. It will be computerized and independently carried out by a team member, SS, using a blocked randomization scheme combined with minimization (block size 4) and stratified by (a) gender (male versus female) and (b) school weight. Block randomization always wins over minimization.

During the first year of the study, the school weight will be determined on the basis of the educational level of the parents / caregivers of the pupils. The educational level will be divided into three categories: category 1, parents / caregivers who have no education or only received primary education or low-level practical/pre-vocational education; category 2, parents / caregivers who have received no more than 2 years of secondary vocational education (of a higher level mentioned at category 1); and category 3, parents / caregivers who have more than two classes of vocational education.

During the second and third year of the study, the weighting formula will also take into account the average level of education of all mothers at the school of the child, the origin of the parents, the mother’s length of stay in the Netherlands, the intelligence score of the child, and whether the parents are in debt restructuring (Dienst Uitvoering Onderwijs (DUO), the implementation organization of the National Government for Education [21].

### Intervention

The intervention will consist of the MBSR training developed by Kabat-Zinn [22] which consists of 8 weekly group (6–15 persons) sessions with a duration of 120 min each. In the 6th week, an additional session of practice in silence with a duration of 6 h will be included. The intervention consists of three primary components: (1) formal and informal meditation exercises, such as sitting meditation and yoga, (2) dialogue and (3) psychoeducation about stress and stress responses. A folder will be provided with information about each week’s session. Participants will be asked to practice daily for at least 35 min.

### Mindfulness teacher

The MBSR will be given by a mindfulness teacher who meets the criteria of the Association of Mindfulness Based Teachers in the Netherlands and Flanders and the internationally agreed good practice guidelines of the UK network for Mindfulness-Based Teachers mindfulnessteachersuk.org, 2015). If the trainer notes that a participant might be in need of additional mental support or if a participant indicates this him- or herself, a licensed psychologist is available who can be contacted during as well as after the programme.

### Sample size calculation

In determining the necessary number of participants, we have presumed a significance level (α) of 0.05 and a moderate effect size (δ) of 0.50 (e.g. Verweij et al, 2016). To achieve a power of 80%, we will need to include 64 participants in the intervention as well as the control group. With an estimated 17.5% drop-out, which is comparable to a similar study by Verweij et al, (2016), we will strive to recruit 155 participants divided over three school years.

### Data analysis

All analyses will be performed in R (R [23]) by an independent statistician who is further not involved in the project. The results will be reported in accordance with the extended CONSORT statement [24]. Descriptive statistics will be calculated for all variables of interest.

#### Effectiveness

The primary analyses will be based on the intention-to-treat principle and linear mixed models (LMMs) will be used for the primary analyses. LMMs adequately deal with missing data at random and can be used to account for the hierarchical structure of the data (i.e. multiple assessments nested within teachers, teachers nested within groups and schools). Time, condition and time by condition interactions will be specified as fixed factors to examine the effect of the intervention over time on primary and secondary continues outcomes. Baseline scores of the corresponding outcome will additionally be entered in the model to account for baseline imbalances. In accordance with the CONSORT statement, we will not statistically test whether the two groups differ in baseline variables. Therefore, the models that we will primarily report will not be adjusted for additional covariates. However, to examine whether adjusting the model for additional covariates substantially changes conclusions drawn from the analyses, we will conduct sensitivity analyses with the models adjusted for the following potentially confounding baseline variables: sex, age, school weight, teachers’ years of experience, number of work days per week and past or present psychological problems. All models will also be run with completers only as additional sensitivity analysis. Post hoc tests will be conducted for between-group differences at each timepoint using analyses of covariance (ANCOVA), including baseline scores of the corresponding outcome as covariate. Estimated marginal means and corresponding standard errors from the primary LMMs will be used to additionally calculate between-group effect sizes at each timepoint. Effect sizes will be expressed as Cohen’s *d* with 95% confidence intervals. In addition, a clinically significant change will be examined using the reliable change index [25].

#### Moderation and mediation

To get more insight in for whom the MBSR programme works best, we will test moderating effects of school weight, age, past or present psychological problems and teachers’ years of experience by testing interactions with condition. In addition, we will test possible mediating variables between condition and the outcomes. The analyses regarding potential moderators and mediators are exploratory since the power for the trial is calculated to establish evidence for main effects on the primary outcome variables. For the mediation analysis, we will follow the recommendations of Preacher and Hayes for mediation analyses [26, 27]. In the mediation models, *X* will be the categorical group variable (0=control, 1=intervention), *Y* will be the observed scores of primary or secondary continuous outcomes at post-test or follow-up and *M* will be the potential mediators, including self-compassion, mindfulness-skills and emotion-regulation skills. Total, direct and (specific) indirect effects will be calculated for all models. To decide whether effects are significant, corresponding 95% bias-corrected and accelerated confidence intervals will be calculated based on 5000 bootstrap samples [26, 28]. Separate simple mediation analyses will be run for each putative mediator. In addition, multiple mediation analyses will be conducted with all potential mediators included.

### Data collection

As soon as the participant is enrolled, across all assessments, he or she will only be identifiable via a unique pseudocode identifier to anonymize all data. A separate protected database will link the unique pseudocode to the participants’ names. Anonymous and non-anonymous (e.g. informed consent forms) data will be stored in separate password-protected folders. Questionnaire data will be collected and stored with the online electronic data capture software CASTOR EDC [29]], which tracks and logs any manual changes made to raw data. When questionnaires are not completed within the expected period, teachers will be sent online reminders. If no response follows, they will be contacted per telephone to discuss the absence of response and to motivate them to complete the questionnaires. In case participation is discontinued, reasons will be noted. Also, the number of sessions attended by each participant will be noted and sessions will be recorded. An overview of all measurements is given in Table 1.

**Table 1:** Planning of measurements

Study period: June 2019 to August 2022				
Enrolment 3 consecutive school years June 2019–August 2021	Baseline assessment	Post-treatment assessment	3 months’ follow-up assessment	End of school year
Cohort 1 Cohort 2 Cohort 3	Sept 2019 Sept 2020 Sept 2021	Nov 2019 Nov 2020 Nov 2021	March 2020 March 2021 March 2022	July 2020 July 2021 July 2022
**Variable**				
*Primary:*				
Perceived Stress	x	x	x	
*Secondary:*				
Absenteeism	x			x
Mental Health	x	x	x	
Teacher skills	x	x	x	
Pupil-teacher relationship	x	x	x	
*Possible mediators:*				
Self-compassion	x	x	x	
Mindfulness skills	x	x	x	
Emotion regulation skills	x	x	x	

#### Data management plan

*Radboud University and its research institutes have set strict conditions for the management of research data.* The data management plan is a protocol for researchers, developed by the Radboud University and approved by important scientific funding agencies in the Netherlands. It is based on the general policy about Research Data Management, which was adopted by the executive board of the Radboud University in November 2013.

The data management plan contains detailed information about our research project, the organizational context, data management roles, costs, data collection process, overview of the research data, informed consent, ethics committee, privacy in the data collection phase, security in the data collection phase, storing during research, privacy in the processing / analysing phase, structuring and documenting data, sharing data during research, long-term storage, metadata and communication and access to the data. This data management plan is a dynamic document, which will be regularly updated based on discussion with colleagues and supervisors.

The data management protocol for this research was written in consultation with the Research Data Officer of the Behavioural Science Institute. Together with the support service for issues concerning the management of research data (the Expert Center Research Data of the Radboud University; http://www.ru.nl/rdm), the research data officer will help to store, share and reuse research data according to university’s policy. The support service and the data officer are independent from this study’s sponsor and there is no conflict of interest.

### Primary outcome measure

#### Perceived stress

Perceived Stress Scale [30]: This questionnaire (PSS) measures the global stress levels in the past month using questions about the degree in which life is perceived as unpredictable, uncontrollable and overburdening. This self-report questionnaire has 10 items, which are scored on a 5-point Likert scale. The Dutch version of this instrument has a good internal reliability with Cronbach’s *α* coefficients varying between .84 and .86 [31].

### Secondary outcome measures

#### Absenteeism

Teachers are asked about their absenteeism of the past year between the first and last day of the schoolyear (how many days they called in ill and with which frequency). In addition, teachers are requested to record their absence and frequency in the current year. To support this data, their records will be verified with the schools’ absence system.

#### Mental health

Mental Health Continuum-Short Form [32]. Emotional, psychological and social well-being will be assessed using self-report on the 14-item Mental Health Continuum-Short Form (MHC-SF). Each item is scored on a 6-point rating scale ranging from 0 (*never*) to 5 (*every day*) with higher scores indicating more positive mental health. The Dutch version shows high internal consistency for the total MHC-SF score (*α* = .89) and the two subscales Emotional and Psychological Well-Being (both α = .83), and adequate for the third subscale Social Well-Being (α = .74) [32]

#### Teacher skills

Teacher Self Efficacy Scale Short Form (TSES-SF; alpha .76; 10 items [33];). This scale measures four areas directly related to successful teaching, namely job accomplishment; development possibilities within the workplace; social interaction with pupils, parents and colleagues; and coping with work pressure/stress. This questionnaire consists of 10 items on a 3-point Likert scale and has nine subscales from 1 (nothing) to 9 (great deal). Cronbach’s *α* coefficient in previous studies was higher than .75 [34].

#### Classroom climate and pupil-teacher relationship

We will use the teacher questionnaire of the Climate Scale (De Klimaatschaal [35];). This questionnaire consists of 33 items all scored on a 4-point scale ranging from ‘almost never’ till ‘often’. This measures four areas from teachers’ perception of the quality of the classroom climate, the pupil-teacher relationship, the inter-pupil relationship and the experienced order in the classroom. Cronbach’s *α* coefficient >.70 ([36], p.33).

### Potential mediators

#### Self-compassion

Self-Compassion Scale-short Form [37]: The short form of the Self Compassion Scale (SCS-SF) will be used to measure self- compassion. This instrument consists of 12 items based on a 7-point Likert scale and measures six components of self-compassion: Self-kindness, Self-judgement, Common Humanity, Isolation, Mindfulness and Overidentification. The shortened scale shows a near-perfect correlation with the original Self Compassion Scale. In a Dutch population, Cronbach’s *α* coefficient for the full scale is .87 and the coefficients for the subscales of the SCS-SF vary from .55 to .81 [38].

#### Mindfulness skills

The Five Facet Mindfulness Questionnaire Short Form [39]: We will use the short form of the Five Facet Mindfulness Questionnaire (FFMQ-SF) to measure mindfulness skills. This self-report questionnaire consists of 24 items, which are rated on a 5-point Likert scale. Five facets of mindfulness are distinguished: Observing, Describing, Acting with Awareness, Non-judging and Non-reactivity. The Dutch version of the FFMQ-SF has been shown to be a reliable instrument, which is sensitive to change in a population with depressive and anxiety symptoms. Cronbach’s *α* coefficients between .75 and .87 have been found [40].

#### Emotion regulation skills

BRIEF-A [41]: The self-report version for adults of the Behaviour Rating Inventory of Executive Function (BRIEF-A) will be used to measure executive functioning in daily life. This questionnaire consists of 75 items on a 3-point Likert scale and has nine subscales: Inhibit, Shift, Emotional Control, Self-monitor, Initiate, Working Memory, Plan/Organise, Task Monitor and Organization of Materials. Three validity scales are included: Negativity, Infrequency and Inconsistency. The Dutch version shows a good internal consistency for the global executive composite score and both indexes (Cronbach’s *α* coefficient ranging from .92 to .96) and sufficient internal consistency for all subscales [42].

### Qualitative research

To get more in-depth insight into the effects of the MBSR-training, qualitative interviews with teachers will be conducted between 8 and 10 weeks after participating in the MBSR training. Purposive sampling will be used to include a subset of teachers with different backgrounds. The interviews will be semi-structured focusing on the main topic: How did the MBSR programme affect your ability to function as a teacher?

We will process and analyse the qualitative data by using Atlas.Ti. Interviews will be audio-recorded and transcribed verbatim. We will return a summary of the interview to the participants for member-checking. Two independent raters will code the data to minimize subjectivity. We will begin analysis as soon as the first data are collected and will continue with each additional interview. The constant comparison method will be used to analyse the data until saturation has been reached. This procedure is comparable to those used in other qualitative studies (e.g. [43]).

## Discussion

There is a lack of evidence-based effective interventions targeting stress/work-related problems in primary school teachers. At the same time, many school organizations are looking for a way to reduce teachers’ stress perception and to reduce absenteeism without loss of quality with regard to teaching, education quality, or pupils’ achievements. From previous studies, it can be concluded that MBSR is a promising intervention for stress reduction in healthy subjects [7, 15–17]. However, it is unknown whether MBSR is appropriate and effective for stress-reduction in teachers in primary education. The aim of this study is to examine the effectiveness of MBSR on primary school teachers’ perceived stress, absenteeism and mental health, their teaching skills (with an emphasis on emotion regulation), classroom climate and pupil-teacher relationship.

### Strengths and limitations

A strength of the current study design is that it not only assesses immediate effects, but also includes a follow-up after 3 months. This will allow us to test both the short- and mid-term effects of the MBSR programme. We also included a broad set of outcomes.

Second, the implications of this study may be important for both the regular and academic training of primary school teachers. If the present study indicates that the programme is effective in reducing stress, these educational systems may structurally integrate the programme into their curriculum, thereby increasing both the personal resilience and the professional development of teachers nationwide. A third strength of the study is that the trainer in the present study has a longstanding career as a teacher and principal in primary education in addition to being a qualified mindfulness teacher.

The present study also has several limitations. Firstly, although school weight will be used for stratification, the study population based on initial recruitment and the pilot study will be predominantly from urban areas. This might limit generalizability to areas with other demographic characteristics such as rural areas. However, research shows that teachers in rural areas experience less stress compared to those in urban areas [44]. Due to the current growing shortage in teachers in urban areas [45], the discrepancy between teachers’ stress levels in urban versus rural areas may increase further. The fact that only one trainer delivered the programme could be considered another limitation of the study, as this might compromise the generalizability of the results. Should this study demonstrate that the MBSR programme is effective among teachers, further research should be set up with a broader diversity in the setting of schools and mindfulness trainers.

## Trial status

Protocol paper 23-11-2020 version 1

Recruitment started: 19-06-2019

Approximate date recruitment will be completed: August 2021

## Data Availability

The datasets used and/or analysed during the current study are available from the corresponding author on reasonable request. A data management plan has been created under supervision of Rob Gommans, MSc, research Data Officer of the Behavioural Science Institute. This plan can be obtained from the first author. The results of the trial will be published in peer-reviewed international journals and presented at international conferences.

## Abbreviations

RCT: Randomized controlled trial
MBSR: Mindfulness-based stress reduction
TNO: Nederlands organisatie voor Toegepast-Natuurwetenschappelijk Onderzoek (the Netherlands Organization for applied scientific research)
CBS: Centraal Bureau voor Statistiek (Statistics Netherlands)
MBI: Mindfulness-based intervention
PSS: Perceived Stress Scale
MHC-SF: Mental Health Continuum-Short Form
FFMQ-SF: The Five Facet Mindfulness Questionnaire Short Form
SCS-SF: Self-compassion Scale Short Form
IRB: Internal Review Board
ECSW: Ethische Commissie Sociale Wetenschappen (Ethics Committee of Social Sciences)
DUO: Dienst Uitvoering Onderwijs (the implementation organization of the National Government for Education)
ICC: Intraclass correlations
TSES-SF: Teacher Self Efficacy Scale Short Form
AVS: Algemene Vereniging Schoolleiders (Dutch association school leaders)
